# Efficacy and safety of pyrotinib‐containing regimen in the patients with HER2‐positive metastatic breast cancer: A multicenter real‐world study

**DOI:** 10.1002/cam4.5056

**Published:** 2022-07-27

**Authors:** Sha Yin, Yajing Chi, Yangyang Du, Jingfen Wang, Changping Shan, Weiwei Yi, Mao Shang, Xiaochu Man, Qiaorui Tan, Huihui Li

**Affiliations:** ^1^ Department of Breast Medical Oncology, Shandong Cancer Hospital and Institute Shandong First Medical University and Shandong Academy of Medical Sciences Jinan Shandong Province China; ^2^ School of Medicine Nankai University Tianjin China; ^3^ Department the 2nd Ward of Breast Surgery Linyi Cancer Hospital Linyi Shandong Province China; ^4^ Department of Breast and Thyroid Oncology Affiliated Hospital of Jining Medical University Jining Shandong Province China; ^5^ Department of Oncology Shandong Provincial Hospital Affiliated to Shandong First Medical University Jinan Shandong Province China; ^6^ Department of Oncology, Jinan Central Hospital, Cheeloo College of Medicine Shandong University Jinan Shandong Province China

**Keywords:** efficacy, HER2‐positive breast cancer, pyrotinib, real‐world study, safety

## Abstract

**Background:**

Pyrotinib, a novel irreversible epidermal growth factor receptor 2 (EGFR)/HER2 dual tyrosine kinase inhibitor, has shown promising antitumor efficacy with tolerable toxicity in HER2‐positive metastatic breast cancer (MBC) in several clinical trials. However, the clinical trials do not usually well reflect the patients in real clinical settings. Despite several small‐sample studies in real world, the data on pyrotinib as first‐line and third‐or‐later‐line treatment and the efficacy comparison of pyrotinib combined with different regimens are still lacking. Therefore, this study aimed to investigate the efficacy and safety of pyrotinib for the HER2‐positive MBC in real world to replenish more comprehensive data.

**Methods:**

A total of 172 HER2‐positive MBC patients treated with pyrotinib‐based therapy were recruited from multiple centers in nonclinical trial settings from September 2017 to June 2020.

**Results:**

The median progression‐free survival (mPFS) of 172 patients was 8.83 months. The patients, receiving first‐line pyrotinib treatment, had the longest mPFS (20.93 months) compared with those receiving second‐line (8.67 months, *p* = 0.0339) and third‐or‐later‐line (7.13 months, *p* = 0.0075) treatments, respectively. Prior treatment with lapatinib (*p* = 0.012) and site of metastasis (visceral vs. nonvisceral) (*p* = 0.033) were the independent prognostic factors for PFS. The prior treatment with lapatinib compared with lapatinib‐native treatment (5.96 vs. 10.97 months, *p* = 0.0036) and those with visceral metastasis compared with nonvisceral metastasis (8.40 vs. 23.70 months, *p* = 0.0138) had worse mPFS. Among 146 patients evaluated for efficacy, 2.1%, 58.9%, and 32.9% showed complete response, partial response, and stable disease, respectively. Adverse events occurred in 92.4% of the patients with 33.3% Grade 3 and higher adverse events and diarrhea (57.0%), anemia (44.8%), and leukopenia (40.7%) as the most frequent ones.

**Conclusions:**

Pyrotinib‐containing regimen could effectively treat HER2‐positive MBC with acceptable toxicity, including the patients who progressed after lapatinib treatment and with brain metastasis.

## Graphical abstract

Pyrotinib, a novel irreversible epidermal growth factor receptor 2 (EGFR)/HER2 dual tyrosine kinase inhibitor, has shown promising antitumor efficacy and tolerable toxicity in HER2‐positive metastatic breast cancer (MBC) in several clinical trials. Whereas, the clinical trials do not usually well reflect the condition of patients in real clinical settings. Our study showed that the pyrotinib‐based regimen can effectively treat HER2‐positive MBC in real world, including patients, who progressed after lapatinib treatment and with brain metastasis, and drug‐related toxicity are acceptable.

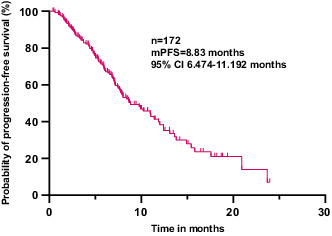

## 1 INTRODUCTION

Breast cancer has the highest incidence and mortality among females with malignant tumors worldwide, which are 24.5% and 15.5%, respectively.^1^ Based on the 2020 report on the Chinese female population, the incidence and mortality of breast cancer were ranked first and fourth, respectively.^2^ It has become an overwhelming threat, endangering women's health and life. Human epidermal growth factor receptor 2 (EGFR/HER2)‐positive breast cancer accounts for 15%–20% of all breast cancers and has a high invasive potential and poor outcome before the emergence of anti‐HER2 therapy.^3^, ^4^ The development of the anti‐HER2 drugs greatly improved the prognosis of these patients. The median overall survival time of the HER2‐positive metastatic breast cancer (MBC) patients can be prolonged to nearly 5 years after the standard double‐target first‐line therapy.^5^ Furthermore, all the HER2‐positive MBC patients develop natural and acquired resistance to anti‐HER2 drugs.^6^ The clinical applications of novel anti‐HER2 therapies are critical for improving the prognosis of HER2‐positive breast cancer.

Pyrotinib is an oral, irreversible, tyrosine kinase inhibitor (TKI) of HER1, HER2, and HER4 that promotes cellular apoptosis and inhibits the proliferation of cancer cells.^7^ It binds competitively to the binding domain of the EGFR family through the homologous structure of intracellular adenosine triphosphate, thereby inhibiting the phosphorylation of tyrosine kinase, which leads to blocking the activation of downstream RAS/RAF/MEK/MAPK and PI3K/AKT signaling pathways and inhibiting the growth of tumor cells.^8^, ^9^ In phase I clinical studies, pyrotinib showed good antitumor effects with acceptable tolerability.^10^, ^11^ The successive phase II and III (PHOEBE) studies, which were strictly designed randomized controlled clinical trials, demonstrated superior combined efficacy of pyrotinib and capecitabine compared with that of lapatinib and capecitabine.^12^, ^13^ Moreover, the PHENIX study further confirmed the efficacy of pyrotinib, especially in patients with brain metastases (BM) at baseline.^14^ However, the strict inclusion criteria for the patients in pyrotinib‐related clinical studies might not well reflect the efficacy and safety of pyrotinib in real clinical settings. Although several real‐world studies have been reported,^15^, ^16^, ^17^, ^18^, ^19^ therapeutic data still need to be supplemented, such as the comparison of pyrotinib's efficacy in combination with different regimens. Therefore, this study aimed to investigate the efficacy and safety of the pyrotinib‐containing regimen in the real world in order to replenish more comprehensive data, especially those not covered in previous studies.

## 2 METHODS

### 2.1 Study design and patient's eligibility

It was a retrospective, multicenter, and real‐world study in which the patients from Shandong Cancer Hospital and Institute, Shandong Provincial Hospital, Jinan Central Hospital, and Linyi Cancer Hospital were recruited from September 2017 to June 2020. The study strictly followed the Declaration of Helsinki guidelines.

Eligibility criteria for the recruitment of patients were as follows: (1) ≥18 years of age; (2) histologically or cytologically confirmed MBC; (3) HER2‐positive, diagnosed as 3+ using immunohistochemistry staining or positive using fluorescence in‐situ hybridization; (4) treated with pyrotinib‐based regimen from September 2017 to June 2020 but did not participate in any clinical trials; (5) at least one measurable lesion according to Response Evaluation Criteria in Solid Tumors (RECIST 1.1) criteria; (6) estimated survival time ≥12 weeks; and (7) complete and available medical records.

All the patients signed a written informed consent before participating in this study. Electronic medical records were consulted to retrieve clinical data and the characteristics of included patients. The disease stage at initial diagnosis was determined according to the 8th edition of the American Joint Committee on Cancer (AJCC) TNM staging system (2017).^20^ And all the data collected from the four hospitals were administrated by Shandong Cancer Hospital and Institute.

### 2.2 Treatment and follow‐up

The standard usage and dosage of pyrotinib used for the treatment of patients was 400 mg orally once a day. The actual pyrotinib dosage and treatment regimen of pyrotinib monotherapy or combination therapy was determined by the clinician according to the clinical conditions of the patients and their wishes. The treatment was discontinued until the emergence of progressive disease (PD) or intolerable treatment‐related toxicities.

The patients, for whom, the treatment was terminated for non‐PD or nondeath reasons, were followed up for efficacy evaluation until PD, initiation of other antitumor therapy or death, or whichever occurred first. All the patients were followed for survival calculation until PD, death, or whichever occurred first. For safety assessment, the patients were followed up until 28 days after the last administration of pyrotinib.

### 2.3 Efficacy and safety evaluation

All the target lesions were measured at baseline using computed tomography (CT) or magnetic resonance imaging (MRI) and repeated every two to three cycles. The efficacy was evaluated according to the RECIST 1.1 criteria. The endpoint of the primary study was progression‐free survival (PFS), which was defined as the time interval between the initiation of pyrotinib therapy and the confirmation of disease progression using a CT/MRI scan or death from any cause. The secondary study endpoints included objective response rate (ORR) and safety. The safety evaluation was based on the National Cancer Institute Common Terminology Criteria for Adverse Events (AEs) (CTCAE 5.0). Trastuzumab resistance referred to disease progression observed in the first imaging evaluation within 3 months or 8–12 weeks after first‐line trastuzumab treatment for MBC or new recurrences diagnosed within 12 months after trastuzumab adjuvant therapy. Trastuzumab refractoriness was defined as disease progression following two and more lines of trastuzumab‐containing regimens after initial remission or disease stabilization at the first radiographic assessment.

### 2.4 Statistical analyses

All the data were analyzed using SPSS 22.0 or GraphPad Prism 7.0 software. The median PFS and 95% confidence interval (CI) were calculated using the Kaplan–Meier method. The comparison between subgroups was performed using a log‐rank test. Univariate and multivariate analyses were performed using Cox regression models. The statistical significance was defined as bilateral *p* < 0.05.

## 3 RESULTS

### 3.1 Baseline and treatment characteristics

From September 2017 to June 2020, a total of 199 patients were enrolled in this study, among which, 27 patients were excluded due to loss of follow‐up before the first efficacy evaluation. Finally, a total of 172 patients were included in the final analysis. The baseline characteristics of patients are listed in Table 1. The age of patients ranged from 31 to 79 years with a median age of 51 years. Invasive ductal carcinoma was observed in 133 patients. Approximately two‐thirds of the patients were hormone receptor (HR)‐positive. Visceral metastases were present among 82% of the patients, where bone (45.9%), lung (43.6%), and liver (42.4%) were the first three most common metastatic sites. Besides, 48 patients (27.9%) developed BM as well. The majority of patients (92.4%) had more than two metastatic sites.

**TABLE 1:** The baseline characteristics of patients

Baseline characteristics	Patients *n* (%) (*n* = 172)
Age	
Median (range), years	51 (30–79)
<65 years	156 (90.7)
≥65 years	16 (9.3)
Histopathologic diagnosis	
Invasive ductal carcinoma	133 (77.3)
Other	39 (22.7)
Hormone receptor status	
HR positive	112 (65.1)
HR negative	60 (34.9)
HER2 status	
2+	32 (18.6)
3+	140 (81.4)
ECOG performance status	
0–1	169 (98.3)
≥2	3 (1.7)
Metastatic sites	
Visceral	141 (82.0)
Nonvisceral	31 (18.0)
Lymph nodes	112 (65.1)
Bone	79 (45.9)
Lung	75 (43.6)
Liver	73 (42.4)
Brain	48 (27.9)
Local recurrence	44 (25.6)
Pleura	14 (8.1)
Adrenal gland	7 (4.1)
Meninges	5 (2.9)
TNM stage at initial diagnosis	
I	12 (7.0)
II	40 (23.3)
III	72 (41.9)
IV	37 (21.5)
Unknown	11 (6.4)
Disease‐free interval (months)	
0	37 (21.5)
≤12	33 (19.2)
>12	100 (58.1)
Unknown	2 (1.2)
Number of metastatic sites	
1	12 (7.0)
2	1 (0.6)
≥3	159 (92.4)

Abbreviation: HR, hormone receptor.

The treatment characteristics are listed in Table 2. Only 16 patients received pyrotinib monotherapy, whereas the rest of the patients received combination therapy of pyrotinib combined with chemotherapeutic agents (capecitabine, vinorelbine, gemcitabine, etc.), trastuzumab, or endocrine agents. A total of 168 patients had previously received HER2‐targeted therapy, including trastuzumab (164, 95.3%), lapatinib (44, 25.6%), pertuzumab (3, 1.7%), trastuzumab emtansine (2, 1.2%), etc. A total of 28, 62, and 82 patients received pyrotinib in the first‐line, second‐line, and third‐or‐later‐line treatment in the advanced setting, respectively. Most of the patients (98.8%) were administered pyrotinib at an initial standard dose of 400 mg/day, whereas the remaining patients (*n* = 2) started pyrotinib at a dose of 320 and 240 mg/day, respectively. During the treatment, 15 patients underwent dose reduction and two patients had treatment interruption.

**TABLE 2:** The treatment characteristics of patients

Treatment characteristics	Patients *n* (%) (*n* = 172)
Previous anti‐HER2 treatment	
Neoadjuvant setting	10 (5.8)
Adjuvant setting	48 (27.9)
Metastatic setting	137 (79.7)
No	4 (2.3)
Prior HER2‐targeted therapy	
Trastuzumab	164 (95.3)
Pertuzumab	3 (1.7)
Lapatinib	44 (25.6)
T‐DM1	2 (1.2)
Other	4 (2.3)
Resistant to trastuzumab therapy	
Resistance	50 (29.1)
Refractoriness	92 (53.5)
No	30 (17.4)
Treatment lines for pyrotinib at metastatic setting	
1	28 (16.3)
2	62 (36.0)
≥3	82 (47.7)
Initial dose of pyrotinib (mg)	
400	170 (98.8)
320	1 (0.6)
240	1 (0.6)
Dose adjustment of pyrotinib (mg)	
400 → 320	12 (7.0)
400 → 320 → 240	1 (0.6)
400 → 240	1 (0.6)
400 → 240 → 400	1 (0.6)
Treatment interruption	
Due to AEs	3 (1.7)
Other	1 (0.6)
Pyrotinib treatment regimen	
Pyrotinib alone	16 (9.3)
Pyrotinib + trastuzumab + chemotherapy	9 (5.2)
Pyrotinib + trastuzumab + vinorelbine	2 (1.2)
Pyrotinib + trastuzumab + capecitabine	4 (2.3)
Pyrotinib + trastuzumab + taxane	3 (1.7)
Pyrotinib + chemotherapy	133 (77.3)
Pyrotinib + taxane	19 (11.0)
Pyrotinib + capecitabine	70 (40.7)
Pyrotinib + vinorelbine	28 (16.3)
Pyrotinib + gemcitabine	9 (5.2)
Pyrotinib + other	7 (4.1)
Pyrotinib + endocrinotherapy	14 (8.1)

### 3.2 Efficacy

**FIGURE 1:**
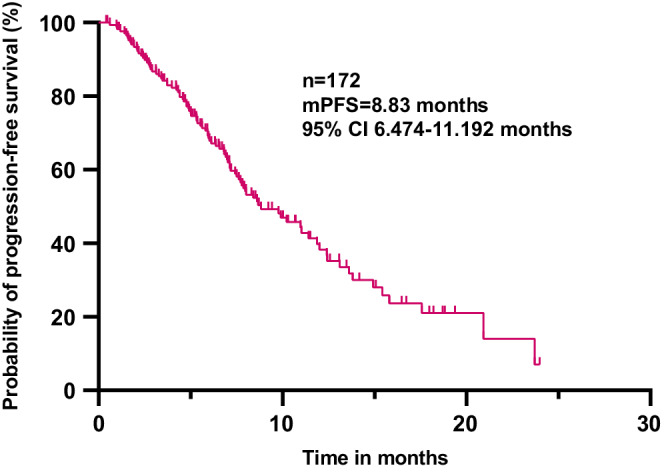
Progression‐free survival of HER2‐positive MBC patients treated with pyrotinib‐contained regimen (*n* = 172). MBC, metastatic breast cancer.

**FIGURE 2:**
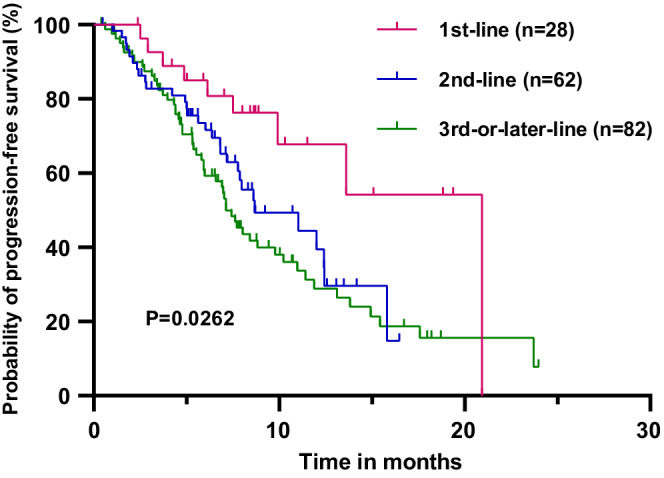
Progression‐free survival of patients in the first‐line (*n* = 28), second‐line (*n* = 62), and third‐or‐later‐line (*n* = 82) pyrotinib‐contained treatment.

**FIGURE 3:**
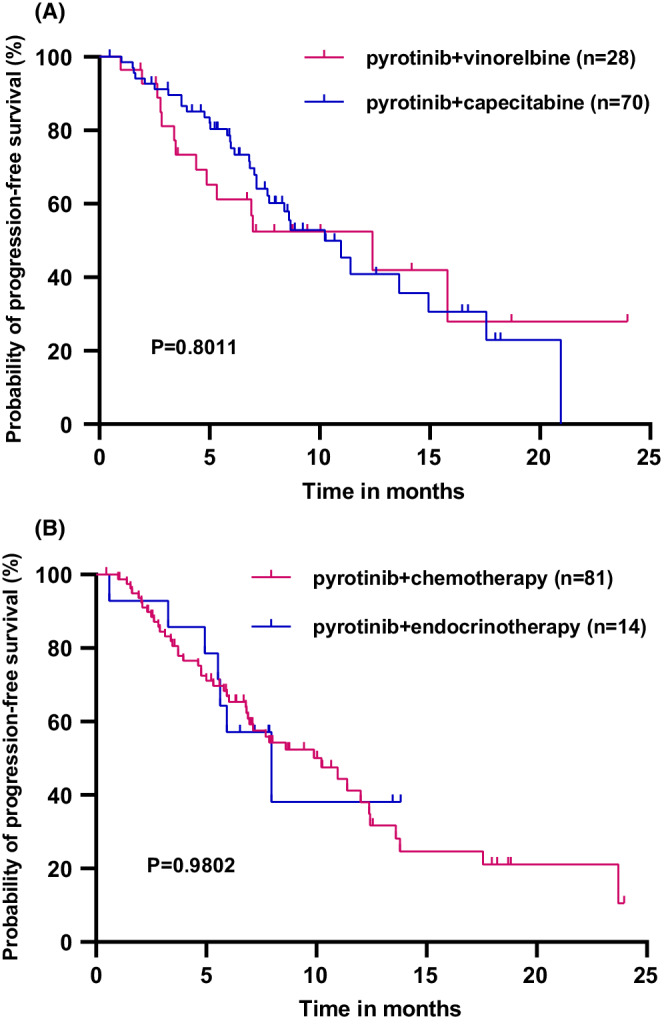
(A) Progression‐free survival of the patients with pyrotinib combined with capecitabine (*n* = 70) and pyrotinib combined with vinorelbine (*n* = 28); (B) Progression‐free survival of HR‐/HER2‐positive patients with pyrotinib combined with chemotherapy (*n* = 81) and pyrotinib combined with endorinotherapy (*n* = 14).

**FIGURE 4:**
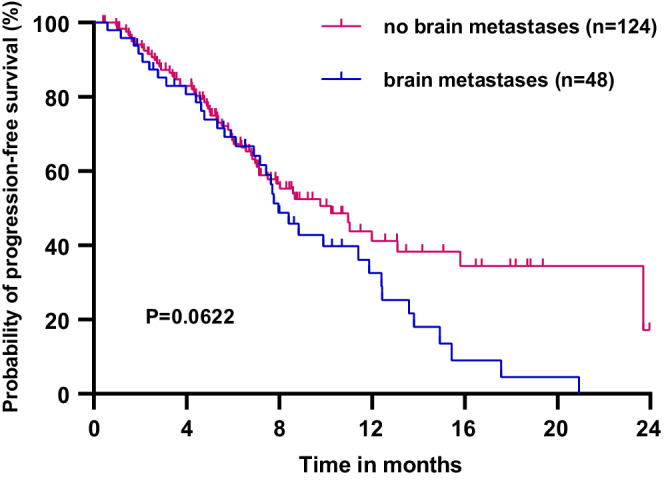
Progression‐free survival of patients with (*n* = 48) and without (*n* = 124) brain metastases.

All the patients in this study were eventually included in the PFS analysis. With a median follow‐up interval of 9.43 months, the median PFS (mPFS) time of 172 patients was 8.83 (95% CI 6.47–11.19) months (Figure 1). The patients, receiving first‐line pyrotinib treatment, had the longest mPFS time compared with those, receiving second‐line and third‐or‐later‐line (20.93, vs. 8.67 vs. 7.13 months, *p* = 0.0262) (Figure 2). The patients treated with the combination of pyrotinib capecitabine and vinorelbine reached the mPFS times of 10.233 and 12.40 months, respectively (*p* = 0.8011) (Figure 3A). Moreover, the mPFS times of HR‐/HER2‐positive patients, who received pyrotinib combined with chemotherapy and endocrinotherapy, were 10.23 and 7.96 months, respectively (*p* = 0.9802) (Figure 3B). The difference in the mPFS times of the patients with and without brain metastasis was not significant (7.97 vs. 10.23 months, *p* = 0.0622) (Figure 4).

A total of 146 patients were evaluated for ORR analysis; the remaining 26 patients were excluded due to lack of measurable lesion or CT/MRI image. Only 2.1% of the patients showed complete response (CR), whereas 58.9% showed partial response (PR) and 32.9% showed stable disease (SD). The efficacy evaluation of intracranial lesions among 44 patients with BM (4 patients were excluded due to lack of measurable lesion or CT/MRI image), 2.1% of the patients showed CR, 56.3% showed PR, and 22.9% showed SD (Table 3).

**TABLE 3:** Best response of patients to pyrotinib

Best response	All patients, *n* (%) (*n* = 172)	Patients with brain metastases, *n* (%) (*n* = 48)
Complete response	3 (2.1)	1 (2.1)
Partial response	86 (58.9)	27 (56.3)
Stable disease	48 (32.9)	11 (22.9)
Progressive disease	9 (6.2)	5 (12.2)
Unknown	26 (15.1)	4 (8.3)
Objective response rate, *n* (%)	89 (61.0)	28 (58.4)

**FIGURE 5:**
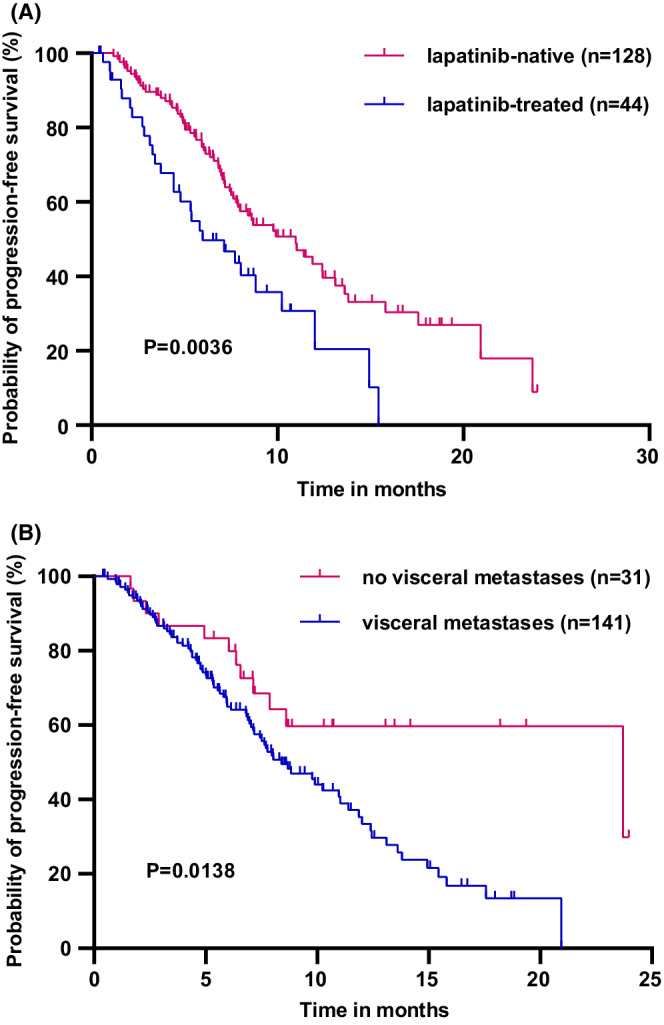
(A) Progression‐free survival of patients prior treated with (*n* = 44) and without (*n* = 128) lapatinib; (B) Progression‐free survival of patients with (*n* = 141) and without (*n* = 31) visceral metastases.

The results of univariate and multivariate analyses are presented in Table 4. The univariate analysis showed that the prior treatment with lapatinib (*p* = 0.0036), treatment with pyrotinib at metastatic settings (*p* = 0.0262), site of metastasis (visceral vs. nonvisceral) (*p* = 0.0138), and liver metastases (*p* = 0.0151) were significantly correlated with PFS. However, the Cox multivariable regression analysis showed that only prior treatment with lapatinib (*p* = 0.012) and site of metastasis (visceral vs. nonvisceral) (*p* = 0.033) were the independent prognostic factors for PFS. Kaplan–Meier curves showed that the patients with prior treatments of lapatinib had worse mPFS than the lapatinib‐native patients (5.96 vs. 10.97 months, *p* = 0.0036) (Figure 5A). The patients with visceral metastasis also had a worse survival compared with the nonvisceral metastasis patients (8.40 vs. 23.70 months, *p* = 0.0138) (Figure 5B).

**TABLE 4:** Log‐rank and Cox multivariate analyses of factors associated with PFS of patients

Factors	HR (95% CI)	Log‐rank analysis *p* value	HR (95% CI)	Cox multivariate analysis *p* value
Age group (<65 vs. ≥65 years)	0.8109 (0.3971–1.656)	0.5292		
DFI (≤12 vs. >12 months)	1.463 (0.8458–2.530)	0.1330		
Hormone receptor status (HR+ vs. HR−)	1.286 (0.8412–1.965)	0.2621		
HER2 status (HER2+ vs. HER2 overexpression)	1.537 (0.8482–2.786)	0.0954	1.504 (0.881–2.566)	0.134
Prior treated by lapatinib (yes vs. no)	**1.895 (1.123–3.198)**	**0.0036**	**1.786 (1.134–2.810)**	**0.012**
Treatment lines for pyrotinib at metastatic setting (1 vs. 2 vs. ≥3)		**0.0262**		0.092
1 vs. 2	**0.4657 (0.2448–0.8857)**	**0.0339**		
1 vs. ≥3	**0.3971 (0.2311–0.6822)**	**0.0075**		
≤2 vs. >2	**0.6532 (0.4348–0.9814)**	**0.0388**		
CEA status (CEA+ vs. CEA−)	1.366 (0.8076–2.310)	0.2235		
CA153 status (CA153+ vs. CA153−)	1.338 (0.8090–2.213)	0.2450		
Number of metastatic sites (≤2 vs. >2)	0.4753 (0.2091–1.081)	0.1906	0.870 (0.251–3.021)	0.827
Site of metastasis (visceral vs. nonvisceral)	**2.062 (1.276–3.333)**	**0.0138**	**2.008 (1.057–3.812)**	**0.033**
Brain metastases (yes vs. no)	1.484 (0.9477–2.323)	0.0622	1.259 (0.803–1.973)	0.316
Lung metastases (yes vs. no)	1.046 (0.6928–1.579)	0.8290		
Liver metastases (yes vs. no)	**1.634 (1.074–2.487)**	**0.0151**	1.195 (0.760–1.880)	0.440
Types of trastuzumab resistance (primary vs. secondary)	0.9747 (0.6109–1.555)	0.9141		
TP53 status (TP53+ vs. TP53−)	0.7480 (0.3814–1.467)	0.3624		

Entries in bold represent statistically significant.

Abbreviations: CI, confidence interval; HR, hormone receptor; PFS, progression‐free survival.

### 3.3 Safety

All the grades of AEs and grade 3/4 AEs are summarized in Table 5 according to the patients' self‐reports, laboratory tests data, and medical records. Given that the present study was retrospective, the omission of AEs was inevitable. AEs of any grade occurred in 92.4% of the patients, Grade 3 and higher AEs were observed in 33.3% of the whole study cohort. Diarrhea was the most frequent AE (57.0%) followed by anemia (44.8%) and leukopenia (40.7%). No treatment‐related death was observed. Grade 3/4 AEs were not observed in 81.3% of patients who received pyrotinib alone. Furthermore, the incidence of the most AEs in the pyrotinib combination group was relatively higher compared with the monotherapy group. In short, the pyrotinib‐related toxicities were manageable and acceptable.

**TABLE 5:** Adverse events of patients

Adverse event	All patients (*n* = 172)		Pyrotinib alone (*n* = 16)		Pyrotinib + other drugs (*n* = 156)	
	Any grade *n* (%)	Grade 3–4 *n* (%)	Any grade *n* (%)	Grade 3–4 *n* (%)	Any grade *n* (%)	Grade 3–4 *n* (%)
None	13 (7.6)	115 (66.7)	0	13 (81.3)	13 (8.3)	102 (65.4)
Diarrhea	98 (57.0)	27 (15.7)	10 (6.3)	2 (12.5)	88 (56.4)	25 (16.0)
Anemia	77 (44.8)	14 (8.1)	7 (43.8)	1 (6.3)	70 (44.9)	13 (8.3)
Leukopenia	70 (40.7)	11 (6.4)	2 (12.5)	0	68 (43.6)	11 (7.1)
Neutropenia	52 (30.2)	14 (8.1)	1 (6.3)	0	51 (32.7)	14 (9.0)
Hypertriglyceridemia	47 (27.3)	0	2 (12.5)	0	45 (28.8)	0
Nausea	43 (25.0)	1 (0.6)	3 (18.8)	0	40 (25.6)	1 (0.6)
Increased AST	39 (22.7)	2 (1.2)	3 (18.8)	0	36 (23.1)	2 (1.3)
Increased ALT	31 (18.0)	1 (0.6)	4 (25.0)	0	27 (17.3)	1 (0.6)
Vomiting	28 (16.3)	1 (0.6)	2 (12.5)	0	26 (16.7)	1 (0.6)
Stomatitis	23 (13.4)	0	2 (12.5)	0	21 (13.5)	0
Lymphopenia	22 (12.8)	2 (1.2)	1 (6.3)	0	21 (13.5)	2 (1.3)
Thrombopenia	16 (9.3)	3 (1.7)	2 (12.5)	0	14 (9.0)	3 (1.9)
Rash	12 (7.0)	2 (1.2)	2 (12.5)	0	10 (6.4)	2 (1.3)
Asthenia	12 (7.0)	0	1 (6.3)	0	11 (7.1)	0
Hand‐foot syndrome	11 (6.4)	2 (1.2)	2 (12.5)	0	9 (5.8)	2 (1.3)
Hyperbilirubinemia	8 (4.7)	0	0	0	8 (5.1)	0

## 4 DISCUSSION

The current standard treatments for the HER2‐positive MBC include trastuzumab combined with pertuzumab and docetaxel regimen from the CLEOPATRA trial for the first‐line setting and T‐DM1 from the EMILIA trial for the second‐line setting.^21^, ^22^ Unfortunately, both the T‐DM1 and pertuzumab have not been covered by insurance for HER2‐positive MBC in China yet. The novel anti‐HER2 agents, which have emerged in recent years, such as DS‐8201, neratinib, tucatinib, etc., have greatly improved the prognosis of HER2‐positive MBC,^23^, ^24^, ^25^ but they are unavailable in China. As a small‐molecule and self‐developed TKI, pyrotinib was approved in the second‐line treatment for HER2‐positive MBC due to good results in previous clinical trials in China.^12^, ^13^, ^14^ Furthermore, many studies, which have evaluated the efficacy and safety of other pyrotinib‐containing regimens in the first‐line treatment for HER2‐positive MBC and (neo)adjuvant therapy for early or locally advanced HER2‐positive breast cancer, have obtained satisfactory preliminary results.^26^, ^27^, ^28^, ^29^ Several discrepancies are generally observed in efficacy and safety between the clinical trials and real‐world studies. Several small‐sample or single‐center studies have attempted to explore the real‐world results of pyrotinib.^15^, ^16^, ^17^, ^18^, ^19^ The present study further complemented current real‐world data and minimized the gap in the efficacy and safety of pyrotinib in clinical trials in an additional different population. This study provided varying pyrotinib‐containing patterns for HER2‐positive MBC to the confusing context of HER2‐targeted therapy.

The mPFS of the patients in this study was 8.83 months and the ORR was 61.0%, which were inferior to the data of the pyrotinib group in PHOEBE (mPFS, 11.1 months; ORR, 68.6%) and PHENIX (mPFS, 12.5 months; ORR, 67.2%) studies.^13^, ^14^ This might be attributed to several differences in the patients' conditions in the real world compared with clinical trials. The patients in clinical trials previously received up to two lines of treatment in recurrent and/or metastatic settings, whereas, in the present study, 47.7% of patients were previously treated with at least three lines of treatment, 97.6% of the patients received anti‐HER2 therapy before pyrotinib, 82.0% of the patients suffered from visceral metastasis, and 92.4% of the patients had more than three metastasis foci, thereby showing a more complex and intractable population. Several previous real‐world studies included 72, 97, 122, and 168 patients and showed mPFS times of 7.6, 7.8, 6.3, and 8.07 months and ORRs of 26.4%, 34.3%, 29.5%, and 40.47%, respectively,^15^, ^16^, ^18^, ^19^ which were slightly worse than those reported in the current study. The patients, who received first‐line pyrotinib treatment, had longer mPFS times compared with those, who received second‐line (8.67 months) and third‐or‐later‐line (7.13 months) in the present study (*p* = 0.0262); these results were consistent with those obtained by Li et al.^17^ This suggested that the earlier use of pyrotinib might be beneficial for the patients. However, it still needs rigorous randomized controlled trials to verify the efficacy of pyrotinib in the first‐line treatment of HER2‐positive MBC.

Since T‐DM1 was not available for the advanced patients in China until June 2021, lapatinib‐based therapy was still the primary second‐line treatment regimen for the patients included in this study. Although lapatinib and pyrotinib are both small‐molecule TKI drugs, pyrotinib can irreversibly and simultaneously inhibit HER1, HER2, and HER4, which is different from lapatinib's reversible inhibition of HER1 and HER2.^9^ The PFS of pyrotinib combined with capecitabine was significantly longer than that of the lapatinib combined with capecitabine (12.5 vs. 6.8 months, *p* < 0.0001) in the PHOEBE study. Furthermore, both the current study and real‐world data by Lin et al. demonstrated that lapatinib‐native patients could benefit more from pyrotinib than lapatinib‐treated patients.^18^ T‐DM1 has been used as the standard second‐line treatment for HER2‐positive MBC, which is the mainstream in the world. A head‐to‐head trial, comparing the T‐DM1‐ and pyrotinib‐contained regimens, is eagerly anticipated. However, a meta‐analysis, including 12 randomized controlled trials, such as PHENIX, PHOEBE, GBG26, KATE2, EMILIA, EGF100151, Cameron, Pivot, Martin, etc., aimed to compare and rank the efficacy of the current six anti‐HER2 treatment regimens.^30^ The result revealed that the combination therapy of pyrotinib and capecitabine was the most likely option to improve PFS in the patients after treatment with trastuzumab. In addition, pyrotinib is more easily available and affordable than the other novel anti‐HER2 agents. Pyrotinib combined with capecitabine has been recommended as a preferred 2nd‐line treatment regimen for the HER2‐positive MBC by the Chinese Society of Clinical Oncology (CSCO) guideline. Li et al. also explored the efficacy and safety of pyrotinib combined with vinorelbine in the real world; the results showed an mPFS of 7.8 months and an ORR of 34.3%.^19^ The current study further compared pyrotinib combined with capecitabine and pyrotinib combined with vinorelbine and showed no statistical difference in the PFS (10.23 vs. 12.4 months, *p* = 0.8011). The potential of pyrotinib combined with vinorelbine or other chemotherapy regimens is worthy of further exploration.

The use of anti‐HER2 combined with chemotherapy or endocrinotherapy as the preferred option for HR‐positive and HER2‐positive MBC is still controversial. The efficacy of dual blockade of HER2 and HR has been *confirmed* in several studies. The ALTERNATIVE study was designed to compare the combination therapy of lapatinib, trastuzumab, and endocrinotherapy with trastuzumab and endocrinotherapy and lapatinib and endocrinotherapy in the second‐line treatment for HR‐positive and HER2‐positive MBC patients, who were previously treated with trastuzumab; the results showed mPFS of 11, 5.7, and 8.3 months, respectively.^31^ This implied that the HR‐positive and HER2‐positive MBC patients could achieve good efficacy by using the dual‐HER2 blockade combined with endocrinotherapy in the second‐line treatment. In the monarcHER study, CDK4/6 inhibitor combined with trastuzumab and fulvestrant showed a survival advantage of a few months over trastuzumab combined with chemotherapy for the HER2‐positive MBC patients, who experienced trastuzumab failures. Additionally, the EGF30008 study emphasized that lapatinib combined with letrozole significantly prolonged PFS (8.2 vs. 3.0 months) and reduced the risk of recurrence by 29% compared with the letrozole group in the first‐line treatment for HR‐positive and HER2‐positive MBC.^32^ Similarly, the TAnDEM and eLEcTRA studies demonstrated that the anti‐HER2‐targeted therapy combined with an aromatase inhibitor (AI) significantly prolonged the PFS as the first‐line treatment for HR‐positive and HER2‐positive MBC patients compared with the AI alone.^33^, ^34^ Moreover, the PERTAIN study also observed that the PFS of first‐line pertuzumab combined with trastuzumab and AI was superior to that of trastuzumab combined with AI.^35^ The SYSUCC‐002 randomized clinical trial presented at the 2021 ASCO meeting showed that for the HR‐positive and HER2‐positive MBC patients, the efficacy of trastuzumab combined with endocrinotherapy was not inferior to that of the trastuzumab combined with chemotherapy with lower toxicity.^36^ In the current study, the mPFS of pyrotinib combined with endocrinotherapy group reached 7.96 months, which further confirmed the efficacy of endocrinotherapy combined with anti‐HER2 therapy for the treatment of HR‐positive and HER2‐positive MBC. The anti‐HER2 therapy is still the basis of treatment for HR‐positive and HER2‐positive breast cancer and the combination of pyrotinib combined with endocrine agents might be explored in the future.

About 25%–50% of the HER2‐positive MBC patients still develop BM. Fortunately, the anti‐HER2 systemic therapy can improve the prognosis of HER2‐positive MBC patients.^37^ Several studies have shown consistent improvements in PFS in patients with BM treated with macromolecular anti‐HER2 drugs. For example, the CLEOPATRA study showed that pertuzumab combined with trastuzumab and docetaxel delayed the occurrence of BM; the patients with BM tended to have a better survival.^38^ Moreover, in the DESTINY‐Breast 01 study, DS‐8201 reached the mPFS of 18.1 months in the patients with BM and the median overall survival of patients with CNS metastasis treated with T‐DM1 in the EMILIA study was better compared with the control group (26.8 vs. 12.9 months, *p* = 0.0081).^23^, ^39^ The small‐molecule anti‐HER2 drugs for HER2‐positive MBC patients with BM have similar effectiveness. The HER2CLIMB study showed that the tucatinib group reduced the intracranial progression of HER2‐positive MBC compared with the placebo group (mPFS 7.6 vs. 5.4 months, *p* < 0.001) and also reduced the risk of death to nearly half.^25^ In addition, the NALA and TBCRC022 studies also confirmed the efficacy of small‐molecule TKI drugs for patients with HER2‐positive breast cancer and BM.^24^, ^40^ In the PHENIX study, the pyrotinib group showed a longer mPFS in the HER2‐positive MBC patients with BM compared with the placebo group (6.9 vs. 4.2 months, *p* = 0.011).^14^ As reported in the PERMEATE study, the CNS‐ORR of pyrotinib combined with capecitabine in breast cancer patients with BM reached 76.9%, further confirming the effectiveness of pyrotinib in the patients with HER2‐positive breast cancer and BM.^41^ A total of 48 patients with BM were enrolled in the present study, with an mPFS of 7.97 months and an ORR of 58.4%; these results were comparable to the efficacy of other small‐molecule anti‐HER2 drugs against CNS cancer in HER2‐positive patients reported in the previous real‐world studies.^15^, ^16^, ^17^, ^18^ None of the patients with BM underwent surgery but 16 of them received cranial radiotherapy, which might have offered better control of BM lesions. The mPFS (11.87 vs. 7.7 months, *p* = 0.4958), ORR (78.57% vs. 70.37%, *p* = 0.849), and CNS‐ORR (78.57% vs. 51.85%, *p* = 0.185) of the patients with BM treated with radiotherapy were better than that of those, who were not treated with radiotherapy in the present study, although no difference in treatment approaches was observed. This was consistent with the previous reports by Chen et al. and Lin et al.^15^, ^18^ The above data suggested that the addition of local radiotherapy to pyrotinib‐based therapy might be more beneficial for the HER2‐positive MBC patients with BM.

In the present study, pyrotinib‐based therapy was well tolerated and the most common AE and grade 3/4 AE was diarrhea, which was consistent with the previous findings but the incidence was lower than those.^15^, ^16^, ^17^, ^18^ This might be due to the vast clinical experience of clinicians with the widespread use of pyrotinib in clinical settings. Moreover, the prophylactic and timely application of loperamide hydrochloride and the adjustment of patients' dietary structure also greatly reduced the incidence and severity of diarrhea. In addition, the observation of AEs depended on the self‐report of patients to some extent, which might have caused the omission of some AEs.

There are several limitations to this study. The first limitation is its retrospective nature and the probable existence of selection bias. In addition, the sample size was relatively small in this study, especially in certain subgroup analyses, such as only 16% of patients received pyrotinib in the first‐line setting and only 25% of the patients had prior lapatinib, which resulted in low statistical power. Furthermore, other studies have yielded results similar to this study; however, the present study still has new findings and differences by contrast. First, the analysis complemented the existing evidence from an additional 172 patients, which is the largest multicenter retrospective study of pyrotinib to date. Compared with the single‐center studies, such as that reported by Li et al.,^17^ this study reached less selection bias and more credible conclusions. Second, the patients in this study had better representation and generalizability of the real world, with more complicated characteristics, including more visceral metastases and varying combined regimens. Thirdly, for the first time, this study compared the efficacy of pyrotinib combined with capecitabine or vinorelbine to explore the possibility of pyrotinib combination with other chemotherapeutic agents. This study also evaluated the combined efficacy of pyrotinib with chemo‐ or endocrine therapy to provide alternative chemotherapy regimens for the HR‐positive and HER2‐positive MBC. Finally, in distinction from prior any real‐world study, the AEs were described for pyrotinib monotherapy in this study.

## 5 CONCLUSION

The pyrotinib‐based regimen could effectively treat the patients with HER2‐positive MBC, including those, who progressed after lapatinib treatment or with BM, and showed acceptable drug‐related toxicities. Moreover, the efficacy of pyrotinib combined with different regimens, especially as the first‐line therapeutic regimen, is worth exploring in future clinical trials, such as pyrotinib combined with vinorelbine or endocrinotherapy for the HR‐positive and HER2‐positive MBC or combined with cranial radiotherapy for BM.

## AUTHOR CONTRIBUTIONS

Conception/design: Sha Yin and Yajing Chi. Provision of study material or patients: Yangyang Du, Jingfen Wang, Weiwei Yi, Mao Shang, and Huihui Li. Collection and/or assembly of data: Changping Shan, Xiaochu Man, and Qiaorui Tan. Data analysis and interpretation: Sha Yin and Yajing Chi. Manuscript writing: Sha Yin and Yajing Chi. Final approval of manuscript: Huihui Li.

## CONFLICT OF INTEREST

The authors declared that they have no conflict of interest.

## ETHICAL APPROVAL STATEMENT

This study was approved by the Ethics Committee of Shandong Cancer Hospital (SDTHEC2020005004).

## CLINICAL TRIAL REGISTRATION NUMBER

This study was registered on the Chinese Clinical Trial Registry (ChiCTR2000038163).

## Data Availability

There are no restrictions on data availability.
